# Local 3D matrix confinement determines division axis through cell shape

**DOI:** 10.18632/oncotarget.5848

**Published:** 2015-10-15

**Authors:** Lijuan He, Weitong Chen, Pei-Hsun Wu, Angela Jimenez, Bin Sheng Wong, Angela San, Konstantinos Konstantopoulos, Denis Wirtz

**Affiliations:** ^1^ Department of Chemical and Biomolecular Engineering, The Johns Hopkins University, Baltimore, Maryland 21218, USA; ^2^ Johns Hopkins Physical Sciences - Oncology Center, The Johns Hopkins University, Baltimore, Maryland 21218, USA; ^3^ Departments of Oncology and Pathology and Sidney Kimmel Comprehensive Cancer Center, The Johns Hopkins University School of Medicine, Baltimore, Maryland 21205, USA

**Keywords:** 3D matrix, elongated cell division, long-axis rule, matrix confinement

## Abstract

How the division axis is determined in mammalian cells embedded in three-dimensional (3D) matrices remains elusive, despite that many types of cells divide in 3D environments. Cells on two-dimensional (2D) substrates typically round up completely to divide. Here, we show that in 3D collagen matrices, mammalian cells such as HT1080 human fibrosarcoma and MDA-MB-231 breast cancer cells exhibit division modes distinct from their Counterparts on 2D substrates, with a markedly higher fraction of cells remaining highly elongated through mitosis in 3D matrices. The long axis of elongated mitotic cells accurately predicts the division axis, independently of matrix density and cell-matrix interactions. This 3D-specific elongated division mode is determined by the local confinement produced by the matrix and the ability of cells to protrude and locally remodel the matrix via β1 integrin. Elongated division is readily recapitulated using collagen-coated microfabricated channels. Cells depleted of β1 integrin still divide in the elongated mode in microchannels, suggesting that 3D confinement is sufficient to induce the elongated cell-division phenotype.

## INTRODUCTION

The orientation of cell division, which positions the daughter cells relative to embryonic axis, is regulated in numerous developing tissues such as epithelial sheets, kidney tubules, elongation of the avian primitive streak, and shaping of the neural plate in avians and mouse [1–3]. Misorientation of the mitotic spindle or the cell division direction is implicated in multiple diseases, including tumor development [4, 5]. Cell shape is thought to determine the direction of cell division in several systems, i.e., cells divide along an axis perpendicular to their major axis, which is established during interphase or early prophase. This is referred as the “long-axis rule” proposed by Oskar Hertwig [6]. This rule has been demonstrated in model systems such as yeast, invertebrate animals including sea urchin [7, 8], embryos of zebrafish [9], amphibians, fish [10], xenopus [11], and *Caenorhabditis elegans* [12].

Two-dimensional (2D) matrix-coated dishes constitute one of the most common model systems for investigating mammalian cell division [13–17]. However, many types of mammalian cells divide in three-dimensional (3D) matrices, including metastatic cancer cells in the stromal/interstitial 3D extracellular matrix, cancer cells at secondary metastatic sites, human and mouse fibroblasts and fibrosarcoma cells located in collagen I-rich 3D connective tissues. Adding a third dimension to the cellular microenvironment by employing a three dimensional (3D) matrix could better recapitulate the microstructure, mechanical properties and biochemical presentation of both normal and pathologic tissues [18–21]. Indeed, cells grown in a 3D matrix exhibit significant differences in differentiation, gene expression, mode of migration and proliferation compared with their counterparts placed on 2D substrates [18–20, 22, 23].

How the axis of mammalian cell division is controlled in 3D environments remains largely unexplored. Single mammalian cells in 2D culture typically round up completely during mitosis. Their cell division orientation is determined by cell shape during interphase, which is “memorized” by the rounded cell through force-sensing retraction fibers that remain connected to the underlying substrate [15]. Whether this long-axis rule also applies to mammalian cell division in 3D microenvironments is unclear. Do single mammalian cells round up into spheres like their counterparts on 2D substrates? Is the cell-division axis determined by cell shape?

To address these questions, we quantitatively investigate cell division in 3D collagen matrices using live-cell imaging assay, time-resolved reflection confocal microscopy, and quantitative imaging analysis. We show that mammalian cells exhibit a division mode in 3D matrices distinct from their counterparts on 2D substrates, with a markedly higher fraction of cells remaining highly elongated through the entire mitotic process. Cells dividing in this elongated mode progress through mitosis without any delay and daughter cells continue to proliferate normally. The orientation of the major axis of these mitotic cells accurately predicts the orientation of the division axis, which is independent of matrix density and cell-matrix interactions. However, local confinement induced by the collagen matrix, produced by the β1-integrin-mediated protrusions of the cells during interphase, is a critical factor determining the fraction of cells undergoing the distinct division phenotype. This elongated mode of cell division can be readily recapitulated using narrow (microfabricated) microchannels, whereas it mostly disappears in wide microchannels. Importantly, all β1-integrin knockdown (KD) cells in the microchannels also divide in the elongated mode, suggesting that a 3D confinement is sufficient for the elongated cell division phenotype. Our results introduce a “long-axis rule” in 3D matrices and reveal novel roles for cell-matrix interactions in regulating cell division modes in 3D environments.

## RESULTS

### Cell shape determines division orientation in 3D collagen

**Figure 1:**
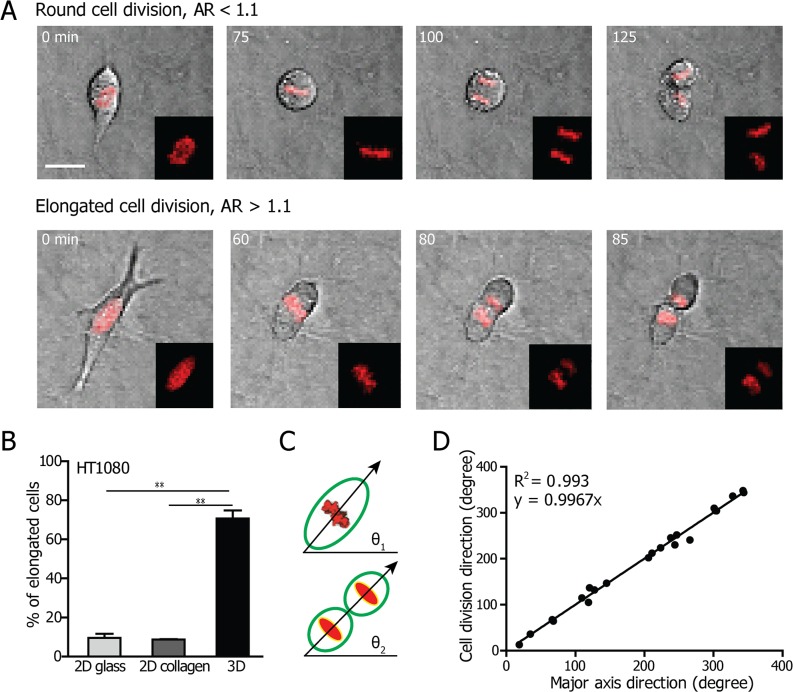
Cell shape determines the division axis of cells in 3D collagen matrices **A.** Representative phase-contrast micrographs of the two cell division modes displayed by HT1080 cells: “round” (defined as cells with cell body fully round up during mitotic phase, upper panel), and “elongated” (defined as cells with cell body remaining elongated through mitotic phase, lower panel). Scale bar, 20 μm. **B.** Percentage of HT1080 cells displaying an elongated morphology during mitosis on 2D glass with or without collagen I coating, and embedded inside a 2 mg/ml 3D collagen I matrix. *n* = 3. Data are represented as mean ± SEM. **C.** Definitions of the angle made by the longest axis of the mitotic cell relative to a horizontal line right before cytokinesis for the elongated mode of cell division (θ_1_) and the angle of the direction of cell division relative to a horizontal line (θ_2_). Here the direction perpendicular to the division plane is defined as the cell division direction. **D.** Correlation between the direction of the major axis and the direction of division for elongated HT1080 cells embedded in 3D collagen matrices. Each data point represents a cell.

To answer the question whether mammalian cells in 3D matrices round up into spheres during cell division similarly to cells on 2D substrates, we investigated cell division by tracking the time-dependent morphology of mitotic cells over long periods of time. HT1080 human fibrosarcoma and MDA-MB-231 human breast cancer cells were embedded in type I collagen matrices. Type I collagen is the most abundant protein in the human body and in the extracellular matrix (ECM) of connective tissues, and thus has been widely used to investigate how functions of eukaryotic cells are modulated by 3D environments [24–26]. The cells used stably expressed H2B-mcherry, a chromatin marker for cell mitotic studies chosen here to accurately distinguish the different phases of cell division [27, 28]. We utilized live-cell microscopy for over 24-h to monitor the progression of cell morphology during the division process in 3D collagen matrices. Interestingly, the division of fibrosarcoma cells in 3D matrices could be divided into two distinct groups: the “round” mode of cell division (cells feature a round shape during mitosis), and the “elongated” one (cells feature an elongated shape during the entire mitotic phase) (Fig. 1A). The latter mode of cell division is absent or rare for cells on 2D substrates, in which cells typically spread during interphase and detach from the substrate and round up into spheres before division [29–31].

The time-dependent aspect ratio (AR = longest axis/shortest axis of the cell) during cell division was monitored to quantify the occurrence of distinct modes of division (as described under Materials and Methods, Supplemental Fig. 1A). The quantification of the aspect ratio starts from about 2 h before the onset of cytokinesis, i.e. before the separation of the two daughter cells. The smallest aspect ratio of the mitotic cell measured during the 2-h video before cytokinesis was employed to define round (AR <1.1) and elongated (AR >1.1) modes of cell division. The results showed that the majority (>90%) of cells on 2D substrates - with or without collagen coating - adopted a round mode of division (Fig. 1B). In contrast, >75% of HT1080 cells underwent elongated cell division in a 3D matrix, significantly higher than HT1080 cells on 2D substrates (8%). We performed the same morphological analysis for MDA-MB-231 cells (Supplemental Fig. 1B). Here, >25% of cells displayed an elongated cell-division phenotype, compared to <5% of cells on 2D substrates.

We further performed the same live-cell experiments on other types of cells, including mouse embryonic fibroblasts (MEF) cells, cancer-associated fibroblasts (CAFs), human embryonic kidney (HEK) 293T cells, and Chinese hamster ovary (CHO) cells. Interestingly, we found that the fraction of elongated cell division mode was more prominent in mesenchymal cells, including HT1080, MDA-MB-231, MEF and CAFs, compared to non-mesenchymal ones, such as HEK 293T and CHO cells (Supplemental Fig. 1D).

Since a majority of HT1080 cells maintained an elongated cell body during the mitotic phase, we asked whether the orientation of the elongated cells prior to division determined the cell's preferential division axis. To perform such analysis, time-resolved microscopy was employed to measure the angles of the longest axis of the elongated mitotic cell right before cytokinesis (θ_1_ in Fig. 1C) and the cell-division axis (θ_2_ in Fig. 1C). We observed a strong positive correlation (*R*^2^ = 0.993) between the direction of the major axis before cell division and the orientation of the cell-division axis for elongated HT1080 cells (Fig. 1D). The shape of the cell prior to cell division suggests a casual relation: the long axis of the cell determines its cell-division axis. The same results were obtained for MDA-MB-231 cells (*R*^2^ = 0.966; Supplemental Fig. 1C).

A previous study using single-cell sea urchin zygote indicated that the more elongated the cells are during mitotic phase, the better they align the division direction along the long axis [7]. To investigate whether such rule also applied to mammalian cell division in 3D matrices, we quantified the angular difference between the cell division axis and the long axis of the cell at mitotic phase as a function of the aspect ratio of the mitotic cell. We found that, the more elongated the cells were during mitotic phase, the more accurately the cell division orientation could be predicated by the long axis of the mitotic cell (Supplemental Fig. 1E).

Taken together, our results indicate that a wide range of mammalian cells, especially mesenchymal cells, in 3D collagen can remain elongated during cell division and that cell shape determines the orientation of cell division.

### Elongated cell division does not preclude mitotic progression and cell proliferation in 3D matrix

**Figure 2:**
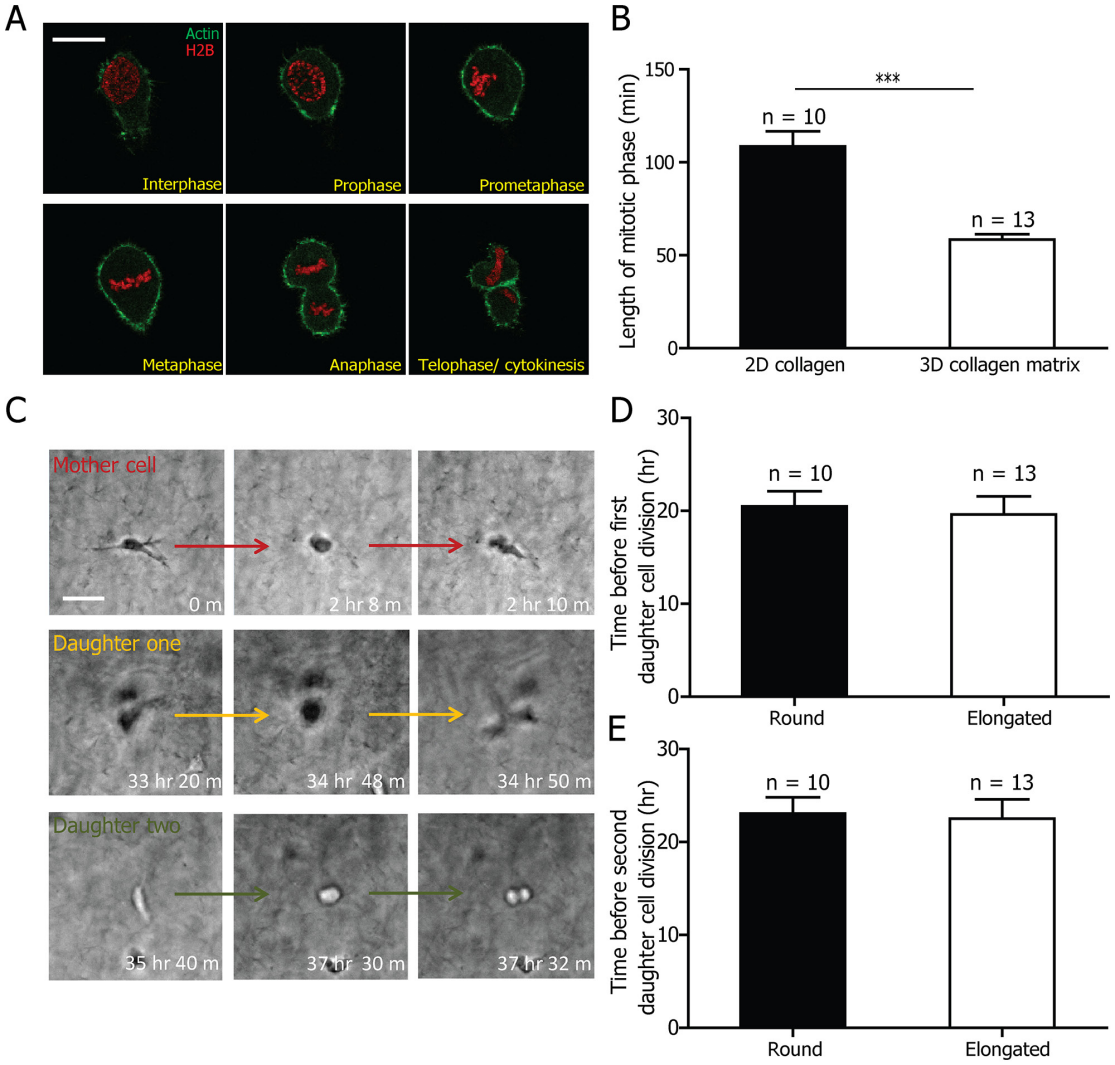
3D-specific cell division phenotype does not interfere with mitotic progression and cell proliferation **A.** Representative micrographs of mitotic progression of an elongated HT1080 cell stably expressing LifeAct-EGFP and H2B-mCherry. **B.** Duration of the mitotic phase of cells in 3D matrix and on 2D substrates. There are 10 cells on 2D substrates and 13 cells in 3D collagen from three independent experiments. **C.** Division of a mother cell that underwent elongated cell division and of its two daughter cells. Red arrow indicates mother cell. Scale bar, 20 μm. **D.** and **E.** Lapse of time before the division of the first (D) and second (E) daughter cell after the mother cell underwent either round or elongated cell division. Differences were not significant. Scale bar, 20 μm. There are 10 round cells and 13 elongated cells from three independent experiments.

Prior studies show that failure of mammalian cells to round up on 2D substrates leads to a delay in mitotic progression [32]. To investigate whether cells in a 3D matrix progressed through mitosis in the elongated division mode, we used confocal microscopy to monitor at high magnification the whole mitotic process of HT1080 cells stably expressing LifeAct-EGFP (to monitor cortical F-actin defining the cell boundary) and H2B-mCherry (to monitor chromosome dynamics; Supplemental Video 1; representative images are shown in Fig. 2A). HT1080 cells in 3D matrices, which divided in the elongated phenotype, progressed through the mitotic phase similarly to their counterparts on 2D substrates. The mitotic phases started with the dissolution of the nuclear membrane (prophase; frame 2, Fig. 2A), the re-organization of the chromosomes (prometaphase: frame 3), the alignment of the chromosomes in the middle of the cell body (metaphase; frame 4), the separation of the chromosomes (anaphase; frame 5) and the reorganization of the chromosomes and nuclear membrane as well as the separation of the bodies of the two daughter cells (telophase/cytokinesis, frame 6). We further measured the duration of the mitotic phase - spanning from prophase to cytokinesis - of cells in a 3D matrix and compared it with the mitotic phase of cells on 2D substrates. Surprisingly, the mitotic phase of the elongated phenotype in 3D was not delayed but was significantly shorter than the normal division mode in 2D (Fig. 2B), which confirmed that the elongated cell division phenotype did not hinder mitotic progression.

To study whether the daughter cells could progress through cell division, and thereby ensure normal proliferation of HT1080 cells in 3D collagen matrices, we performed long-term live-cell imaging (>48 h). We tracked the division of the mother cells and that of the two daughter cells, and compared the division of cells that had undergone round and elongated cell divisions (Fig. 2C and Supplemental Video 2). We measured the time between the division of the mother cell and the division of the two daughter cells. There was no significant difference in the lapse of time before first daughter cell division after the round or elongated mother cells divided (Fig. 2D). The same conclusion applied to the time before the second daughter cell division (Fig. 2E).

Together, the results showed that cells dividing in the elongated mode in a 3D matrix progress through mitotic phase without any delay, and daughter cells continue to divide to ensure proliferation of the cells, suggesting that mammalian cells in 3D matrix do not require rounding up to carry out normal cell division.

### 3D matrix confinement is associated with the elongated cell-division mode

**Figure 3:**
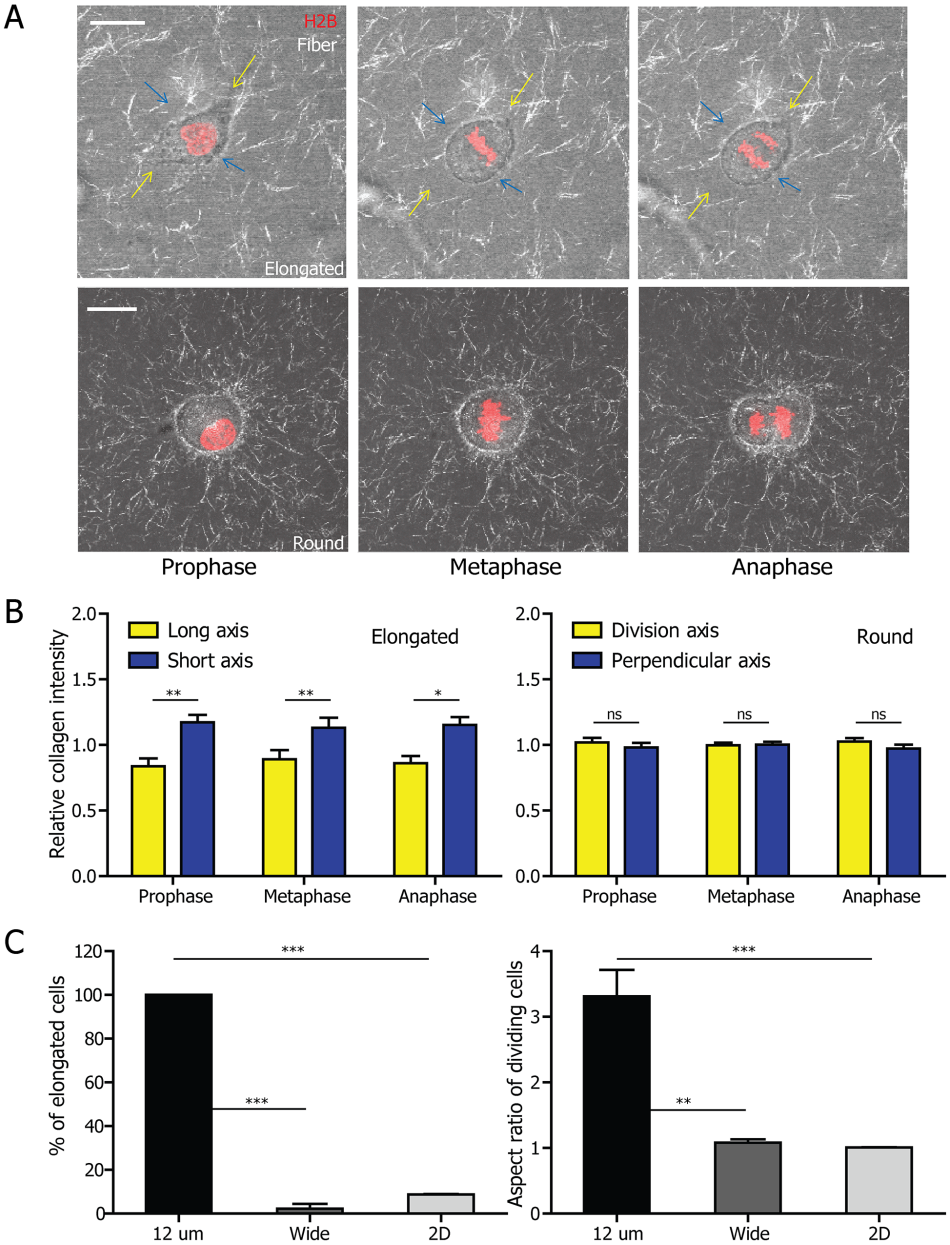
Elongated cell-division mode is associated with 3D matrix confinement and can be recapitulated in microfabricated channels **A.** Representative micrographs obtained from a high-magnification live-cell imaging video of HT1080 cells which undergo elongated (top panel) and round (bottom panel) division. The collagen fibers are visualized by reflection confocal microscopy (RCM). Scale bar, 20 μm. **B.** Quantification of the RCM collagen intensity in the direction of long axis and of the short axis of the elongated (left panel) and round (right panel) dividing cell. *n* = 5 for both elongated and round cells. Data are represented as mean ± SEM. For elongated HT1080 cells, the intensity of collagen fibers in the direction of long axis (indicated by yellow arrows in A) is significantly lower than that in the direction of short axis (blue arrows in A). The RCM intensity of collagen surrounding the round HT1080 cell is the same in those two directions. **C.** The fraction (left panel) of elongated cells and the aspect ratio (right panel) of all dividing cells inside 12 μm-wide and 100–200 μm wide microchannel as well as on 2D substrates. *n* = 3 for both graphs. Data are represented as mean ± SEM. Both the fraction of elongated cells and the aspect ratio of all dividing cells in 12 μm-wide microchannels are significantly higher than for cells in 100–200 μm wide microchannels and on 2D substrate.

We reasoned that the 3D matrix played an important role in the elongated cell division phenotype. To directly investigate how the elongated cell division mode took place in 3D collagen matrices, we combined reflection confocal microscopy (RCM) and fluorescence confocal microscopy to image HT1080 cells stably expressing H2B-mcherry embedded in collagen matrices (Supplemental Video 3). RCM has been applied to non-invasively visualize and quantitate the micro-topography of porous biomaterials prepared from synthetic polymers and 3D collagen matrices [24, 25, 33–37]. During the mitotic phase, from the prophase to anaphase, there are more abundant fibers along the short axis of the cell, with very few fibers - if any - along the longest axis (Fig. 3A, top panel). We further quantified the RCM intensities of the collagen fibers along the long axis and the short axis of the cells at prophase, metaphase and anaphase of the dividing cells. We found that the intensity of collagen matrix surrounding the mitotic cell in the direction of its short axis was significantly higher than that in the direction of the cell's long axis (Fig. 3B, left panel). Using a similar strategy, we monitored the mitotic progression of cells undergoing round division (Fig. 3A, bottom panel). We quantified the intensity of the fibers along the direction of the cell division as well as the direction perpendicular to the cell division axis, since all the axes in the round cells were of the same length. The intensity of the collagen fibers in these two directions surrounding the round cells during mitosis remained similar (Fig. 3B, right panel). This result suggests that the confinement on the short axis of the cell generated by the anisotropic collagen fiber arrangement around the dividing cell is associated with the elongated division phenotype.

To directly assess whether confinement contributed to the elongated cell division phenotype, we employed a “3D-like” collagen-coated microfabricated channel to subject cells to a defined confinement. We fabricated 12 μm-wide microchannels, a width equal to the length of the smallest short axis of the elongated cells in 3D collagen matrix (Supplemental Fig. 2A). HT1080 cells were then allowed to migrate into the microchannels and the cell division process was monitored using live-cell microscopy. We observed that all cells in the narrow microchannels divided in the “elongated” mode (Supplemental Video 4; Fig. 3C). This elongated mode of cell division mostly disappears in wide microchannels of the width of 100–200 μm, as well as on the 2D regions just outside the microchannels. Taken together, these results suggest that a “3D-like” confinement is essential to produce an elongated cell division phenotype.

### Elongated cell-division phenotype depends on interphase cell morphology mediated by β1 integrin

Next we asked how the anisotropic microchannel-like confinement was generated in 3D collagen matrices. In collagen matrices of the same density, different types of cells feature different fractions of cells undergoing elongated division (Supplemental Fig. 1D). We speculated that the cell itself might play a role in the formation of elongated cell shape besides the 3D collagen microenvironment. Thus we examined the interphase morphology of different cell lines, including HT1080, MDA-MB-231, MEF, CAF, HEK 293T and CHO cells. We observed that all these cells could adopt either protruded or round morphologies at interphase in collagen matrices, similar to what has been previously reported for MDA cells [38]. We quantified the fractions of cells undergoing the elongated mode of cell division in 2 mg/ml 3D collagen matrix (Supplemental Fig. 1D). Interestingly, there was a positive correlation between the fraction of cells undergoing elongated cell-division and the fraction of cells with a protruded morphology during interphase, which suggests that the preference for the elongated cell-division phenotype in the 3D matrix scales with the protruded morphology of the cell during interphase (Supplemental Fig. 3A). Invasive cancer cells and fibroblasts are capable of digesting or deforming surrounded matrices by extending protrusions [39–41], through which the spatial confinement of the cells exerted by matrix becomes anisotropic. Based on these observations and previous literature, we hypothesized that the capability of cell to re-organize the matrix through protrusions to create local anisotropic collagen confinement was correlated with the cell's tendency to divide in the elongated division mode.

**Figure 4:**
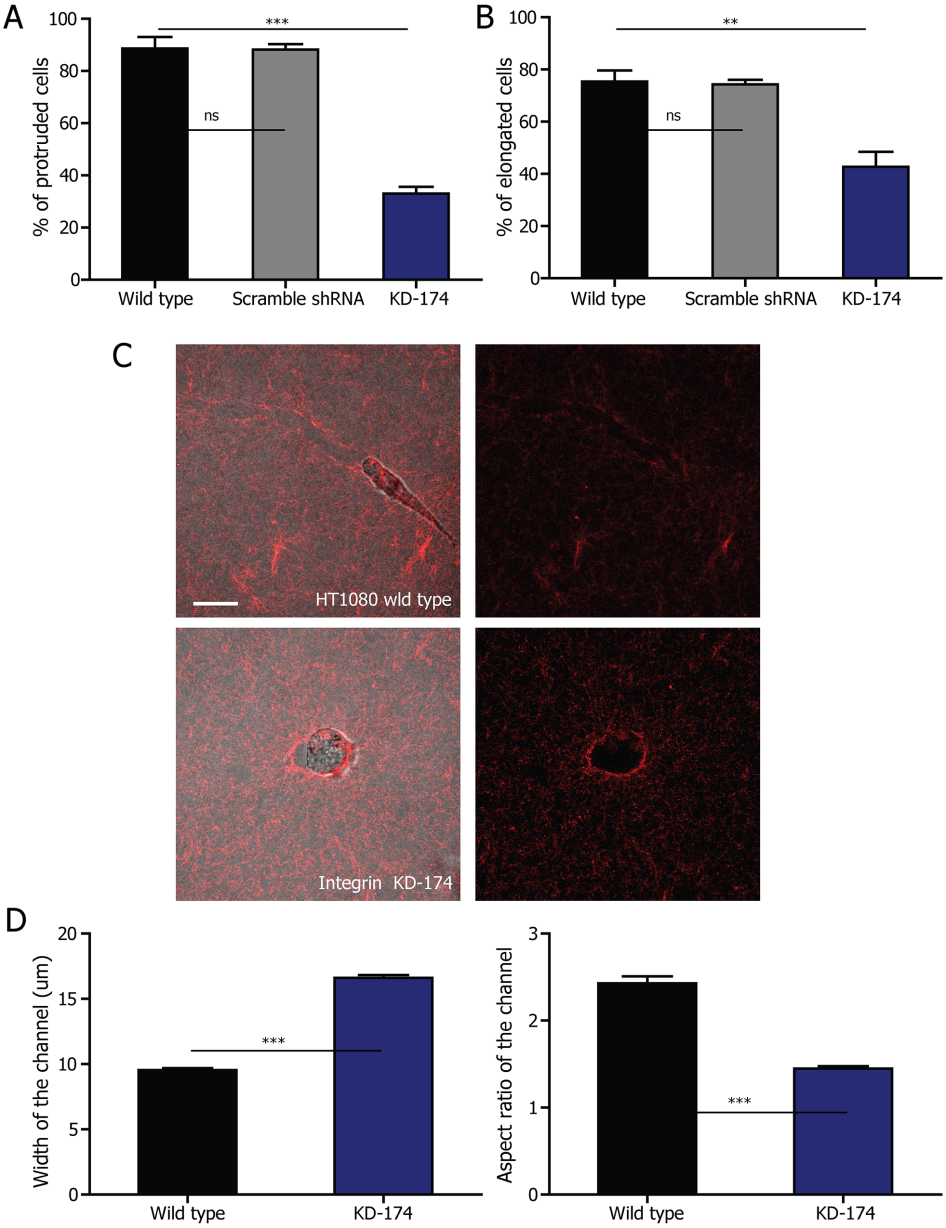
Elongated cell division depends on β1 integrin **A.** Fraction of protruded HT1080 cells during interphase in 2 mg/ml collagen matrix after depletion of β1 integrin. *n* = 3. **B.** Fraction of HT1080 cells undergoing elongated cell division in 2 mg/ml collagen matrix after depletion of β1 integrin. *n* = 3. **C.** Microtracks generated by elongated HT1080 control cells and round β1-integrin-depleted cells are visualized employing fluorescently labeled collagen. Scale bar, 20 μm. **D.** Quantification of the width and aspect ratio of the microtracks generated by the two types of cells in the matrix. The data was analyzed from 387 wild type and 325 β1-integrin KD cells from three independent experiments. All the data are represented as mean ± SEM.

To test this hypothesis, we depleted the adhesion molecule β1 integrin in HT1080 cells and MDA-MB-231 cells through shRNA technology. β1 integrin is a major membrane protein expressed by MDA-MB-231 and HT1080 cells, coordinating cellular interactions with collagen [17, 42, 43]. Thereby, we hypothesized that the depletion of β1 integrin would result in reduced cell protrusion, which would lead to reduced matrix re-organization compared to control cells. The depletion of β1 integrin was confirmed by Western blotting (Supplemental Fig. 3B): the RNAi sequences reduced the expression of β1 ntegrin by >95%, whereas the scramble shRNA did not affect β1 integrin expression. We quantified the fraction of protruded cells in the collagen matrix, which significantly decreased for β1-integrin-depleted HT1080 cells compared to control cells, suggesting that β1 integrin indeed modulated cell morphology at interphase (Fig. 4A). We then quantified the percentage of the different cell division phenotypes in β1-integrin-depleted cells. The percentage of cells undergoing elongated cell division decreased by about 50% (Fig. 4B). The same results were obtained with β1-integrin-depleted MDA-MB-231 cells (Supplemental Fig. 3C and 3D).

We further hypothesized that the failure of β1-integrin-depleted cells to protrude during interphase would prevent cells to remodel the matrix. To test this, we embedded both control and β1-integrin-depleted cells in 2 mg/ml collagen matrices. Indeed, microchannels of the average width of 10 μm, similar to that of the fabricated microchannels (Supplemental Fig. 2A), are generated in collagen matrix by protrusive HT1080 cells. The round β1-integrin KD HT1080 cells, on the other hand, only generate holes of the size similar to or bigger than cells (Fig. 4C and 4D). Using a similar strategy as previously described for HT1080 wild type cells, we combined RCM and fluorescence confocal microscopy to monitor the mitotic progress of a round β1-integrin KD cell. The confinement exerted by the collagen fibers to the non-protruded β1-integrin KD cells before and during mitosis remained isotropic, similarly to the round HT1080 wild type cells (Supplemental Fig. 3E). Taken together, these results suggest that microchannels in the collagen matrix result from cell protrusions regulated by β1 integrin and provide the anisotropic confinement essential to the formation of elongated dividing cells.

To further assess the role of matrix reorganization by protrusive cells, we investigated the matrix reorganization induced by MDA-MB-231 cells and compared it with matrix reorganization induced by HT1080 cells. We found that MDA-MB-231 cells, which have a smaller fraction of protruded interphase cells and elongated mitotic cells, tended to generate holes of the size similar to, or bigger than, the cell diameter, similarly to β1-integrin KD HT1080 cells (Supplemental Fig. 3F). This further validated our hypothesis that the elongated cell-division phenotype depends on the protruded interphase cell morphology, which generates microtracks in the matrix of width smaller than the cell diameter.

Integrins are also involved in cellular response to external mechanical signals [44, 45]. To investigate whether depleting β1 integrin impaired the cellular response to 3D confinement, we introduced β1-integrin KD cells into the microfabricated microchannel as described above. Interestingly, similarly to HT1080 control cells, all the β1-integrin KD cells (*n* = 34) in the microchannel divided in the elongated mode (Supplemental Video 5; Supplemental Fig. 2B). This result suggests that β1-integrin KD cells remain capable of receiving signal from the 3D confinement, which supports the conclusion that it is the failure of β1-integrin KD cells to protrude in collagen during interphase that leads to the decrease in the proportion of elongated-dividing cells. It also shows that β1 integrin is not required for the elongated division phenotype when there is a pre-formed narrow “microchannel”, suggesting that a 3D confinement is sufficient for the elongated cell division phenotype.

### Prediction of the cell-division axis by the elongated cell shape is independent of cell-matrix interaction or matrix density

Retraction of actin fibers from cells plated on fibronectin-coated 2D substrates exerts forces on the mitotic cell body and dictate mitotic spindle orientation and the division axis of Hela cells [15, 42]. In our study, we found that the majority of HT1080 cells (>95% of the dividing cells) and MDA-MB-231 cells (>99% of the dividing cells) withdraw their protrusions before division in 3D collagen matrices. To further determine whether retraction fibers existed for the elongated cells embedded in 3D collagen matrices, we fixed MDA-MB-231 and HT1080 cells stably expressing Life-act-EGFP, and then imaged the cells using confocal microscopy, which is supposed to provide higher magnification and resolution images. Consistent with the low-magnification study, we did not observe residual actin fibers in majority of the elongated mitotic cells in 3D collagen matrices (Supplemental Fig. 4). This observation stimulated us to hypothesize that there was no external force applied by the matrix on the elongated mitotic cells, especially those without residual protrusions.

**Figure 5:**
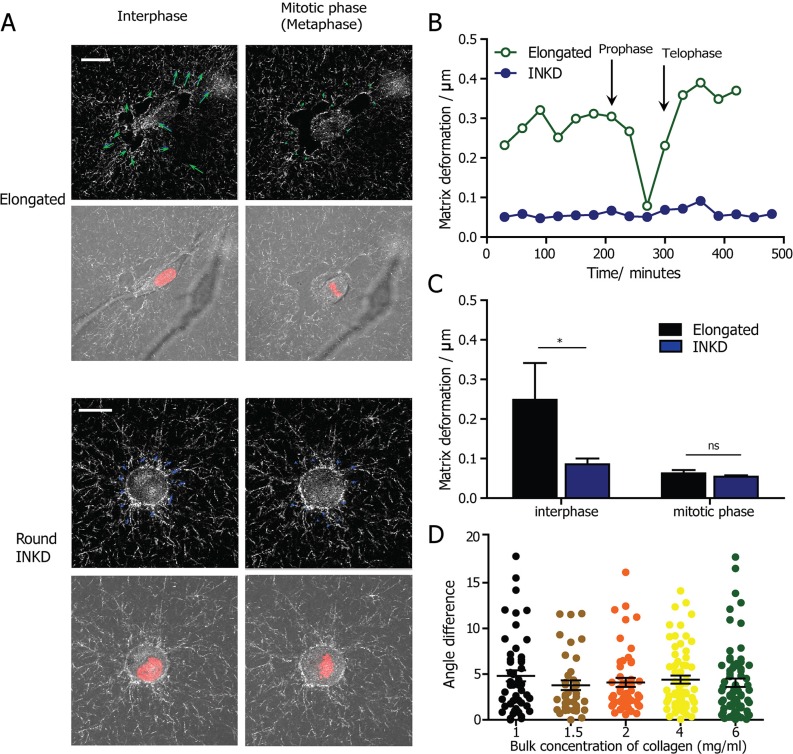
The determination of the cell-division axis by cell shape is independent of cell-matrix interactions and matrix density **A.** Representative micrographs obtained from a high-magnification live-cell imaging video of an elongated HT1080 cell expressing H2B-mCherry, embedded in a collagen matrix. The collagen fibers are visualized by time-dependent reflection confocal microscopy. The deformation vector of the matrix quantified by PIV software was shown in blue arrows. Scale bar, 20 μm. **B.** The change in the magnitude of matrix deformation for matrix-embedded elongated and round β1-integrin-KD cells. The black arrow indicates prophase and telophase. **C.** Quantification of matrix deformation for elongated and round β1-integrin-KD cells during interphase and mitosis, indicating that matrix deformation is minimal during cell division. *n* = 5 for both phases. Data are represented as mean ± SEM. **D.** Comparison of the angle difference between the major axis and the division direction for HT1080 cells undergoing cell division in 3D matrices of varying densities, from 1 mg/ml to 6 mg/ml. The statistical analysis was performed using non-parametric analysis because the data does not pass the normality test.

To examine this hypothesis, we employed time-resolved RCM to monitor the deformation of collagen fibers in real time, which is an indicator of cell-matrix interactions [37]. Combining RCM and live-cell fluorescent imaging, the matrix deformation before, during and after cell division could be visualized (Representative snapshots are shown in Fig. 5A) and quantified using a custom PIV software. We quantified and compared matrix deformation during interphase and mitotic phase for cells undergoing elongated division mode. We observed that the matrix deformation decreased as a cell approached mitosis, and increased after cytokinesis (Fig. 5B). The matrix deformation during cell mitosis is significantly smaller than in the interphase and post-mitotic phases. This result shows that mammalian cells have minimal attachment and interactions with the surrounding matrix when they enter the mitotic phase. We then monitored the matrix deformation by β1-integrin KD HT1080 cells during both interphase and mitotic phase. Depleting β1 integrin significantly reduces the matrix deformation by the cell during interphase. However, there is no difference in matrix deformation during mitotic phase of the round β1-integrin KD cells compared with elongated HT1080 wild type cells (Fig. 5C). Taken together, these results suggest that compared with the round mitotic cell, the elongated mitotic cell does not rely on extra force from the ECM to split the daughter cells along the direction of the major axis of the cell, despite the fact that the mother cell remained elongated before division.

We further investigated whether the prediction of cell-division axis by the major axis was dependent on collagen matrix density; we compared the angular differences between the major axis and the cell division direction of elongated cells embedded in collagen matrices of different bulk densities. Interestingly, there was no difference in the accuracy of predicting cell division direction by the orientation of the major axis (Fig. 5D). This result suggests that the “long-axis rule” holds for cells embedded in 3D matrices and is largely independent of matrix properties.

## DISCUSSION

As one of the most critical events in cellular life, cell division has been investigated for over a century. Little work has probed mammalian cell division orientation in 3D matrices, despite the fact that many types of cells divide in 3D matrices. This might be partly due to the experimental limitations and technical challenges involved in studying cell division in 3D matrices. Previous work on MCF7, U2OS, and HepG2 human cell lines has shown that the proliferation rate of these cells is much lower in 3D matrices than on 2D substrates [46, 47]. Moreover, cell mitosis itself represents a small temporal fraction of the cell cycle [27]. A popular method to facilitate the study of cell division is to synchronize cells using double thymidine blocking [17, 42] or the shake-off method [48, 49], such that a large number of cell division events can be studied simultaneously. However, forced synchronization can have lasting, irreversible effects on cell functions [50]. Cells in a 3D matrix also move in and out of focus, which is another factor limiting the efficiency of capturing cell-division events using high-magnification microscopy.

**Figure 6:**
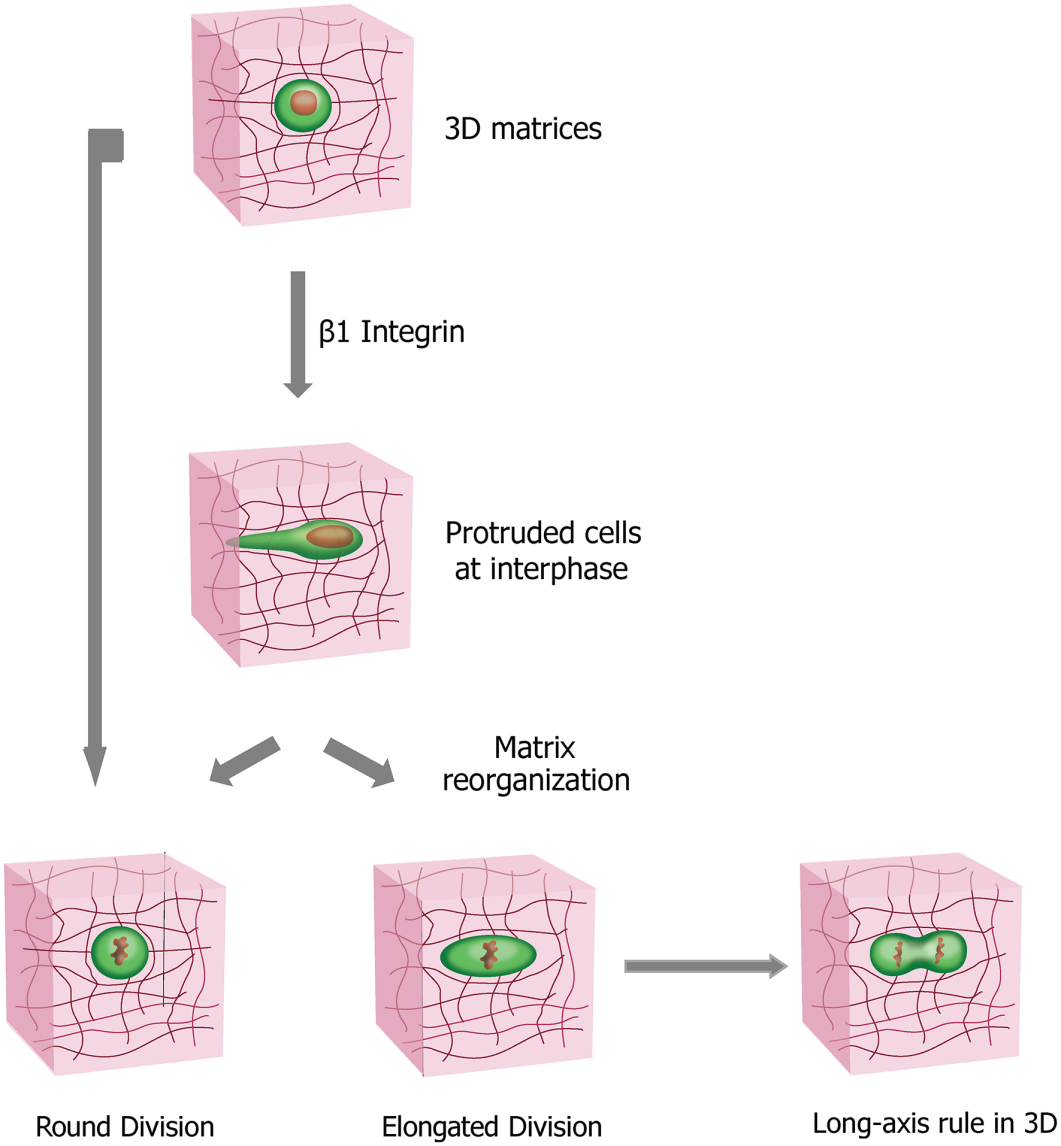
Cell division modes in 3D collagen matrices and the determination of cell division orientation by the direction of the major axis of the mitotic elongated cell Cells exhibit modes of cell division in 3D collagen I matrices distinct from their counterparts on 2D substrates, with a significant higher portion of the cells remaining elongated during division process. The fraction of the cells undergoing the distinct cell division phenotype is synergistically determined by the 3D confinement on mitotic cells exerted by the matrix and the capability of the cells to protrude and to locally remodel the 3D matrix mediated by β1 integrin. The direction of the major axis of the mitotic elongated cell could predict the cell-division axis, a process independent of matrix density and cell-matrix interactions.

Our work presents an extensive study of mammalian cell division in 3D collagen matrices of different densities, integrating live-cell imaging assay, time-resolved reflection confocal microscopy and quantitative imaging analysis. As illustrated in Fig. 6, we show that a significantly high fraction of the cells in 3D collagen matrices remain elongated during mitotic phase in the cell division process. The prediction of the cell-division axis by the elongated cell shape is independent of cell-matrix interactions or matrix density. The percentage of cells undergoing this elongated division phenotype in 3D depends on the synergistic effects between the interphase cell morphology mediated by β1 integrin in the cells and the biophysical properties of matrix, especially the 3D confinement. Protruded cells reorganize the matrix during interphase and create narrow microchannels, such that the mitotic cells are subjected to anisotropic confinement forces exerted by the collagen matrices. Interestingly, this elongated mode of cell division can be recapitulated using narrow microchannels, whereas it mostly disappears in wide microchannels. Moreover, all the β1-integrin KD cells in the narrow microchannels also divide in the elongated mode, suggesting that a 3D confinement is sufficient for the elongated cell division phenotype.

Results from this study shift the previous paradigm of the necessity for cell to round up for proper division on 2D substrate, and reveal for the first time that cells embedded in 3D matrices do not require rounding-up for proper division and proliferation. Furthermore, this study introduces the “long-axis rule” for 3D cell cultures and reveals novel roles for cell-matrix interactions in regulating cell division phenotypes in 3D culture environment.

Mammalian cells cultured on 2D substrates normally round up into a perfect sphere during division. It appears surprising that our results show that both HT1080 and MDA-MB-231 cells can divide normally in the elongated mode. Indeed, recent work by Lancaster et *al*. has shown that a failure of cells to round up not only causes defects in spindle assembly, but also leads to delay in mitotic progression [32]. One factor leading to the apparent contradiction between their study and ours could be the use of a different cell line. Hela cells were employed in the work by Lancaster, whereas we focused on HT1080 and MDA-MB-231 cells. Another difference between the two studies is that they used polyacrylamide hydrogels of calibrated stiffness to flatten cells, whereas we investigated the division of cells fully embedded in 3D collagen matrices. They did not observe obvious defects in the spindle structure in cells growing under moderately stiff gels of 3–15 kPa, which confined cells to a height of 8 μm, roughly half of the unconstrained height of the metaphase plate in control cells. A large proportion of profoundly flattened cells developed multipolar spindles when gel stiffness was further increased or when fixed cell height was reduced below 8 μm. The stiffness of all the collagen matrices in our research is all below 3 kPa [51]. Moreover, the length of the metaphase plate of the elongated HT1080 cells in our study ranges from 12–18 μm, which is about 60–90% of the round cells. Thus, the 3D confinement of cells by the collagen fibers may not be sufficiently strong to lead to a delay in mitotic progression, or a decrease in cell proliferation.

Rounding of cells on planar substrates is a complex process involving the rearrangement of the cortical acto-myosin network, the formation of rigid actin cortex regulated by members of the ERM (ezrin, radixin and moesin) family of actin-binding proteins and localized RhoA activity [29–31, 52]. A more recent study has also suggested that the mitotic rounding is dependent on the role of cells’ ability to regulate osmolarity through ion pumps [53]. Thus, changes in acto-myosin network, actin cortex regulation, RhoA activity or ion pumps might be cell-intrinsic factors contributing to the increased proportions of cells remaining elongated during division process in 3D matrices compared with their counterparts on 2D substrates.

The elongated cell division mode described in this work is reminiscent of cytokinesis B, in which cells do not require the activity of acto-myosin to divide, but rely on cell-substrate adhesion [54–57]. This cell division mode has been observed in certain types of mammalian cells, such as HT1080, on 2D substrates, and drosophila cells in embryos [54–57]. To determine whether the elongated division mode in 3D matrix was cytokinesis B, we monitored cell-matrix interactions by integrating fluorescent imaging and reflection confocal imaging (Fig. 5). We found no significant matrix traction during cell mitosis, suggesting that cell-substrate adhesion was not involved in splitting the two daughter cells of the elongated dividing cells compare with the rounded dividing cells. Furthermore, the division of cells treated with myosin inhibitor blebbistatin was greatly reduced (Supplemental Fig. 5), suggesting that cell division in 3D matrix requires myosin activity. Taken together, these results indicate that the elongated cell division mode in 3D collagen matrix is fundamentally different from cytokinesis B.

Our study focused on the role of cell-matrix interactions in regulating the modes of cell division in 3D. We embedded the cells at low density in the matrix such that the cells did not run into each other during both interphase and mitotic phase. Previous work revealed that the local epithelial topology of neighboring cells can influence the interphase shape of a cell and thereby biases the orientation of the long axis, however, the mitotic cells surrounded by neighbors are still able to fully round up [1]. Future research could investigate the role of cell-cell interactions in the division mode of cells embedded in 3D matrices.

Our results show that the “long-axis rule” applies to mammalian cells in 3D environments: The division direction of the elongated cells is predicted by the orientation of the major axis of the cell during mitosis, which is independent of cell-matrix interactions and matrix properties. The division plane positioning is known to be a complex dynamic process which requires the collaboration between microtubules and actin networks [58–61]. Microtubules center the nucleus and thereby determine the division axis in both sea urchin and fission yeast, for which the division-plane orientation is controlled by cell shape. Work on sea urchin eggs in particular shows that the orientation of cell division plane for any given cell shape is predicted based on microtubule-length dependent forces [7]. Microtubule associated proteins are also involved in orientating cell division direction. The microtubule plus-end-tracking protein EB1 is involved in orientating mitotic spindle of HeLa cells parallel to the 2D substrate, through stabling astral microtubules [17]. Dynein motors, which transport cargos along towards the minus-end of the microtubule, are believed to control spindle positioning by generating forces between actin cortex and the astral microtubules [62]. It will be intriguing to investigate whether microtubules and the associated proteins also play roles in transiting the direction signals from cell shape to the cell division in 3D matrices in the future. Using 2D fibronectin micropattern, Thery *et al*. showed that cell-ECM interactions results in cortical actin heterogeneity, such that the shape of the underlying patterns could dictate the cell-division axis of Hela cells. Future research could focus on investigating whether the distribution of cortical actin in the elongated HT1080 and MDA-MB-231 cells is heterogeneous or not.

HT1080 and MDA-MB-231 exhibit different propensities to be elongated during mitosis in the same collagen matrix. However, HT1080 and MDA-MB-231 cells embedded in Matrigel (Corning Life Sciences) all divide in the round division mode. Matrigel is a gelatinous protein mixture secreted by Engelbreth-Holm-Swarm (EHS) mouse sarcoma cells and resembles the extracellular microenvironment in many tissues. This suggests that different types of cells in different 3D matrices may exhibit different types of division modes. Indeed, recent work published during the preparation of this manuscript reported a study of Swiss 3T3 fibroblast cell division in fibrin gel. They found that 3T3 cells remain long protrusion attached to the matrix during cell division, with cell bodies rounded up [63]. The division direction is correlated with the orientation of the long protrusions, which exert pulling forces to the anchored fibrin matrix during cell division. In our study, the majority of HT1080 cells (>95% of the dividing cells) and MDA-MB-231 cells (>99% of the dividing cells) withdraw their protrusion(s) before division in 3D collagen matrices, and there is no additional pulling forces between cell and matrix during the division of the elongated cells compared with round cells. Future research to unravel the mechanisms controlling the different division modes requires careful and extensive investigation, and will enable the further understanding of mammalian cell division in 3D physiological relevant environment.

## MATERIALS AND METHODS

### Cell culture

Human breast carcinoma cells MDA-MB-231 (Physical Sciences Oncology Center, NIH), human fibrosarcoma HT1080 (ATCC) cells, mouse embryonic fibroblast (MEF, ATCC) and human embryonic kidney 293T (HEK 293T, ATCC) cells were cultured in Dulbecco's modified Eagle's medium (Mediatech Inc.), high glucose (4.5 g/L), supplemented with 10% fetal bovine serum (Hyclone) and 1% Pen/Strep (Sigma). Chinese hamster ovary (CHO, ATCC) Cells were cultured in DMEM, low glucose (1.5 g/L), supplemented with 2 mM L-glutamine (Life technologies), Gibco^®^ MEM Non-Essential Amino Acids (Life technologies), 10% fetal bovine serum (Hyclone) and 1% Pen/Strep (Sigma). Cancer associated fibroblast cells (CAF), kindly provided by Professor Erik Sahai (Cancer research UK London Research Institute, UK), were cultured in Dulbecco's modified Eagle's medium (DMEM, Mediatech Inc.), high glucose (4.5 g/L), supplemented with 10% fetal bovine serum (Hyclone) and 1% ITS (insulin–transferrin–selenium; Invitrogen) [64]. All the cells were maintained in an incubator with 5% CO_2_ at 37°C.

### 2D collagen I-coated substrates

Two-dimensional cell-culture glass-bottom 24-well plates (BD Biosciences) were coated with soluble rat tail type I collagen in acetic acid (BD Biosciences) to achieve a coverage of 60 μg/cm^2^ and incubated at room temperature for 2 h. This concentration was chosen to saturate the surface of the wells. Plates were then washed gently three times with PBS and seeded with cells.

### 3D collagen I matrices

Cells embedded in 3D collagen matrices were prepared by mixing cells suspended in culture medium with soluble rat tail type I collagen in acetic acid (BD Biosciences) to achieve a final concentrations of 1, 1.5, 2, 4 and 6 mg/ml collagen. 1 M NaOH was then added to normalize pH to about 7.0. The mixture was placed in 24-well culture plates (BD Biosciences). Collagen gels solidified within 1 h in an incubator at 37°C and 5% CO_2_, then 500 μl of cell culture medium was added. Cell density in the matrix was kept low to ensure single-cell measurements. Live-cell imaging was conducted 24 h after the addition of inhibitor.

### Stable expression of Life-act-EGFP and H2B-mCherry

The plasmid encoding H2B-mCherry in a lentiviral vector with phosphoglycerate kinase promoter (PGK) was obtained from Addgene (Cambridge, MA, Plasmid 21217). The plasmid encoding Lifeact-EGFP in a lentiviral vector was constructed as previously described [65, 66]. To generate lentivirus particles, HEK293T cells were co-transfected with three plasmids (lentiviral vector, ΔR 8.91, and pMDG-VSVG) using Fugene HD (Promega, Madison, WI). The medium was replaced with fresh medium 24 h after transfection. The lentiviral particles were harvested another 24 h later immediately filtered through 0.45-μm filter (EMD Millipore, Billerica, MA) to remove cellular debris, and then stored at −80°C. For transduction, 1 × 10^5^ cells in a 35-mm culture dish were transduced with lentivirus. Stable expression of H2B-mCherry and Lifeact-EGFP was validated two or three days after transduction using a Nikon TE2000E epifluorescence microscope (Nikon, Tokyo, Japan).

### Depletion of β1 integrin by shRNAs

The shRNA constructs targeting mRNA of β1 integrin were selected using the RNAi design online program from Dharmacon (http://www.dharmacon.com). Two targeting sites were chosen. The sequences used were TGCCTACTTCTGCACGATGT (174) and CCAGCCCATTTAGCTACAAA (674). Successful depletion of the protein was confirmed by Western blotting using the β1-integrin antibodies (BD Pharmingen, 552828) and quantified using ImageJ (NIH).

### Collagen I labeling with TAMRA

Rat tail collagen I was labeled as previously described before [67]. Briefly, TAMRA powder (Life Technologies, Grand Island, NY) was dissolved in DMSO to a final concentration of 10 mg/ml. One ml of high concentrated rat tail collagen I was then injected into a presoaked 10,000 MWCO dialysis cassette (Life Technologies) and dialyzed overnight against 1 L of labeling buffer (0.25 M NaHCO_3_, 0.4 M NaCl). After the collagen was dialyzed, it was mixed with 100 μl of the TAMRA solution diluted in 900 μl of labeling buffer. This collagen/TAMRA solution was then incubated overnight with rotation at 4C° and then dialyzed the next night against 1L of labeling buffer to remove excess dye. Subsequently, this solution was again dialyzed overnight in 1 L of 0.2% (v/v) acetic acid solution, pH 4. The final concentration of TAMRA-labeled collagen was calculated from the measured final volume, and the original volume and collagen concentration.

### Microscope image acquisition

#### Cell aspect ratio during cell division

Images of cells were collected at two-minute intervals using a Cascade 1K CCD camera (Roper Scientific) mounted on a Nikon TE2000E phase contrast microscope equipped with a 10x objective (Nikon) and controlled by NIS-Elemens AR imaging software (Nikon). Custom software was developed in MATLAB to segment cells and to measure their aspect ratio by dividing the length of the longest axis with that of the shortest axis. An average of 80 frames/160 min of the live-cell imaging videos were analyzed for each cell to monitor the change of cell aspect ratios before, during, and after cell division.

#### Quantification of cell division direction and cell major axis orientation

To capture a sufficient number of cell-division events, cells embedded in 3D collagen matrices were imaged at low magnification (10x) at two-minute intervals for 24 h, using a Cascade 1K CCD camera (Roper Scientific) mounted on a Nikon TE2000E phase contrast microscope. NIS-Elemens-AR was used to measure the orientation of the cell major axis before division and the angle of the cell-division axis. Both angles were measured relative to a horizontal line. The angle of the major axis of the cell was defined as the angle between the longest axis of the cell and the horizontal line. The direction of the cell-division axis was measured by the angle between the reference line and a line connecting the center of the two daughter cells.

#### Immunofluorescence microscopy

Fluorescent cells completely embedded inside collagen gels and the TAMRA labelled collagen were imaged ≥150 μm away from the bottom on a Nikon A1 confocal microscope using a 60x water-immersion lens, NA = 1.2, WD = 200 μm (Nikon) and controlled by Nikon Elements imaging software (NIS-3.1). A *z*-step of 0.3 μm was used to optically section the samples for 3D reconstruction.

#### Reflection confocal microscopy for collagen matrices

To visualize collagen fibers in unstained 3D collagen matrices, a Nikon A1 confocal microscope was configured to capture only reflected light (488 nm) from the 488 nm laser used to illuminate the sample and using a 60x water-immersion objective, NA = 1.2, WD = 200 μm (Nikon) and controlled by Nikon Elements imaging software (NIS-3.1). To visualize collagen fibers of the matrix during cell migration and division, a *z*-step of 0.2–1 μm was used to optically section the samples.

### Microfluidic-based microchannel assay to study confined cell division

To physically constrain cell rounding during division, multilayer photolithography and standard replica molding were used to fabricate confining polydimethylsiloxane (PDMS) microchannels, as described previously [68]. The microchannel devices used in this experiment comprised 8000 μm-long and 10 μm–high microchannels of width of 10 μm arrayed perpendicularly between cell seeding and medium supply channels. Masks were designed in AutoCAD (Autodesk, McLean, VA) and transferred to chrome-on-glass darkfield photolithography masks (Photoplot Store, Colorado Springs, CO).

Molds for the microfluidic devices were fabricated using multilayer photolithography. SU-8 3010 (Microchem, Newton, MA) was spun to a thickness of 10 μm on a silicon wafer (University Wafer, South Boston, MA), soft baked, and exposed through a mask defining the 8000 μm-long microchannels using an EVG620 mask aligner (EVG, Austria). The exposed wafer was post-exposure baked, developed with SU-8 developer, and rinsed with isopropanol. To fabricate the cell and medium inlet lines, the photolithography step was repeated using a 50 μm-thick layer of SU-8 3025, with exposure through a mask aligned over the microchannel features. The completed wafer was hard baked for 10 min at 150°C and treated with (tridecafluoro-1,1,2,2,-tetrahydrooctyl)-1-trichlorosilane (Pfaltz & Bauer, Waterbury, CT) overnight to facilitate the release of PDMS from the mold.

The final PDMS microfluidic devices were formed using standard replica molding from the microfabricated silicon wafer. PDMS elastomer and crosslinker (Sylgard 184 kit, Dow Corning, Midland, MI) were mixed at a 10:1 w/w ratio, poured over the wafer, degassed in a vacuum, and cured at 85°C for 2 h. Solidified PDMS were peeled off from the master, punched with a 6 mm diameter hole puncher at the indicated well inlets and outlets and cut to appropriate sizes. PDMS devices and 25 mm × 75 mm microscope slides (Electron Microscopy Sciences, Hartfield, PA) were cleaned with 100% ethanol and blew dried with filtered air, before being treated with oxygen plasma (Plasma Cleaner PDC-32G, Harrick Plasma, Ithaca, NY) for 2 min and 30 s to render the surfaces hydrophilic. The plasma treated PDMS devices were then attached and sealed to the glass slides, forming the eventual 4-walled microchannel devices. To enable cell binding and adhesion, microchannels devices were coated with 20 μg/ml rat tail type 1 collagen (Life Technologies, Frederick, MD) diluted in PBS without magnesium and calcium at 37°C for 1 h followed by 4°C overnight. Right before cell seeding, collagen was aspirated and the microchannels were washed twice with PBS without calcium or magnesium, with each rinse lasting approximately 5 min.

HT1080 or MDA-MB-231 cells were trypsinized with 0.25% trypsin, resuspended in media with 10% FBS to inactivate the action of trypsin and then washed once in media without serum. Cells were then counted and resuspended in serum free media to 2 × 10^6^ cells/ml. 25 μl of the cell suspension (equivalent to a total of 5 × 10^4^ cells) were added to one of the cell inlet wells and allowed about 5 min to position themselves in front of the microchannel entrance and attach. Subsequently, the cell suspension was removed from the cell inlets, and 100 μl of serum-free media was added to each of the 3 bottom right wells and 100 μl of 10% FBS-containing media to the right top most well to create a chemotactic gradient by diffusion across the laminar flow. The devices were incubated at 37°C for 4 to 5 h for the cells to migrate into the channels towards the chemotractant. Once most cells have entered the microchannels, media was aspirated and replenished with 100 μl of 10% FBS containing media in all 6 wells.

Cell division within the microchannels were visualized and recorded via time-lapse live microscopy in an enclosed, humidified microscope staged maintained at 37°C and 5% CO_2_ using software-controlled stage automation (inverted Eclipse Ti microscope; Nikon). Phase contrast images were taken at 7 min interval for a total duration of 24 h with a 10x Ph1 objective. The resultant width of the channels are about 12 μm based on the measurement using NIS-elements (Nikon).

### Quantification of collagen fiber intensity surrounding cell boundaries

Cells stably expressing H2B-mcherry embedded in collagen matrix were imaged using a Nikon A1 confocal microscope with a 60x water-immersion objective, NA = 1.2, WD = 200 μm (Nikon) and controlled by Nikon Elements imaging software (NIS-3.1). The microscope was configured to capture reflected light (488 nm) from the 488 nm laser to collect signal from collagen fibers. H2B-mCherry was visualized using the 561 nm laser. Five z planes at 5 μm intervals at each time point were imaged to ensure the capture of nucleus during possible cell movement in Z-direction. The plane which provides both in-focus images for nucleus and cell boundary was employed for quantification. Boundaries of cells in 3D matrices were first determined and traced manually in the bright field images. The fiber intensity along cell boundaries was calculated based on the reflection confocal microscopy images that were acquired at the same position as cell images. The fiber intensity at each cell boundary pixels are calculated by integrating pixel intensities that within 2 μm extracellular space at the direction. For elongated dividing cells, fiber-mitotic alignment index was calculated using ratio between mean fiber intensity within ± 45° of cell elongation axis to intensity measured from their short axis. For round dividing cells, the index is calculated using the ratio between the mean fiber intensity within ± 45° of the cell division direction and the intensity measured along the direction perpendicular to the cell division axis.

### Collagen network deformation during cell division

The deformation of the collagen matrix in the vicinity of a cell due to the forces exerted by that cell was measured using particle imaging velocimetry (PIV) with sub-pixel resolution [37]. PIV analysis was performed using customized software developed in MATLAB. In brief, to improve accuracy of PIV, anisotropic low pass filtering as described above were applied to enhance the signal of collagen network. An image cross-correlation technique was then used to track the deformation of the collagen network over time at the region of interest. To identify the local displacement of a sub-image region of the collagen network located at (*x*, *y*) from frame k to frame *k* + 1, we extracted a regional window of 15 × 15 pixels centered at (*x*, *y*) at frame *k* and identified the best matching locations (*x*, *y*)*, the locations that feature a maximum normalized cross-correlation coefficient, in the image obtained at frame *k* + 1. The deformation vector was then calculated as the difference (*x*, *y*)* − (*x*, *y*). We monitored the deformation of the collagen network at the same locations over 10 h of observation time. This procedure was performed iteratively over frames to extract the dynamics of network deformation, prior to, during, and after cell division.

### Statistical analysis

All the experiments were repeated at least three times (three distinct biological repeats) for statistical analysis. The mean ± standard error (SE) was determined and statistical analysis was performed with the use of Graphpad Prism (Graphpad Software). Unless stated otherwise, all the errors bars in the figures are standard error of mean (SEM). Normal *T*-test or one-way analysis of variance was conducted to determine the significance of samples with two groups and more than two groups. If the data does not pass the Kolmogorov–Smirnov test, non-parametric test (Kruskal-Wallis test in Graphpad Prism) was employed. The significance of samples with two groups and more than two groups are indicated by the standard Michelin Guide scale (^***^ *p* < 0.001, ^**^ *p* < 0.01, and *p < 0.05). Bonferroni correction was employed for multiple comparisons. Linear regressions were calculated and plotted using Graphpad Prism for correlation plots. Slope and square of the Pearson correlation coefficient (R^2^) of the regression's deviation from zero slope are shown in the plots.

## SUPPLEMENTARY FIGURES AND VIDEOS


