# Supplementary material for: Local 3D matrix confinement determines division axis through cell shape
Source: Oncotarget. 2015 Oct 15;7(6):6994–7011. doi: 10.18632/oncotarget.5848 (PMC4872764; doi:10.18632/oncotarget.5848)
Supplement: Supplementary file 1 [file oncotarget-07-6994-s001.pdf]

SUPPLEMENTARY FIGURES AND VIDEOS

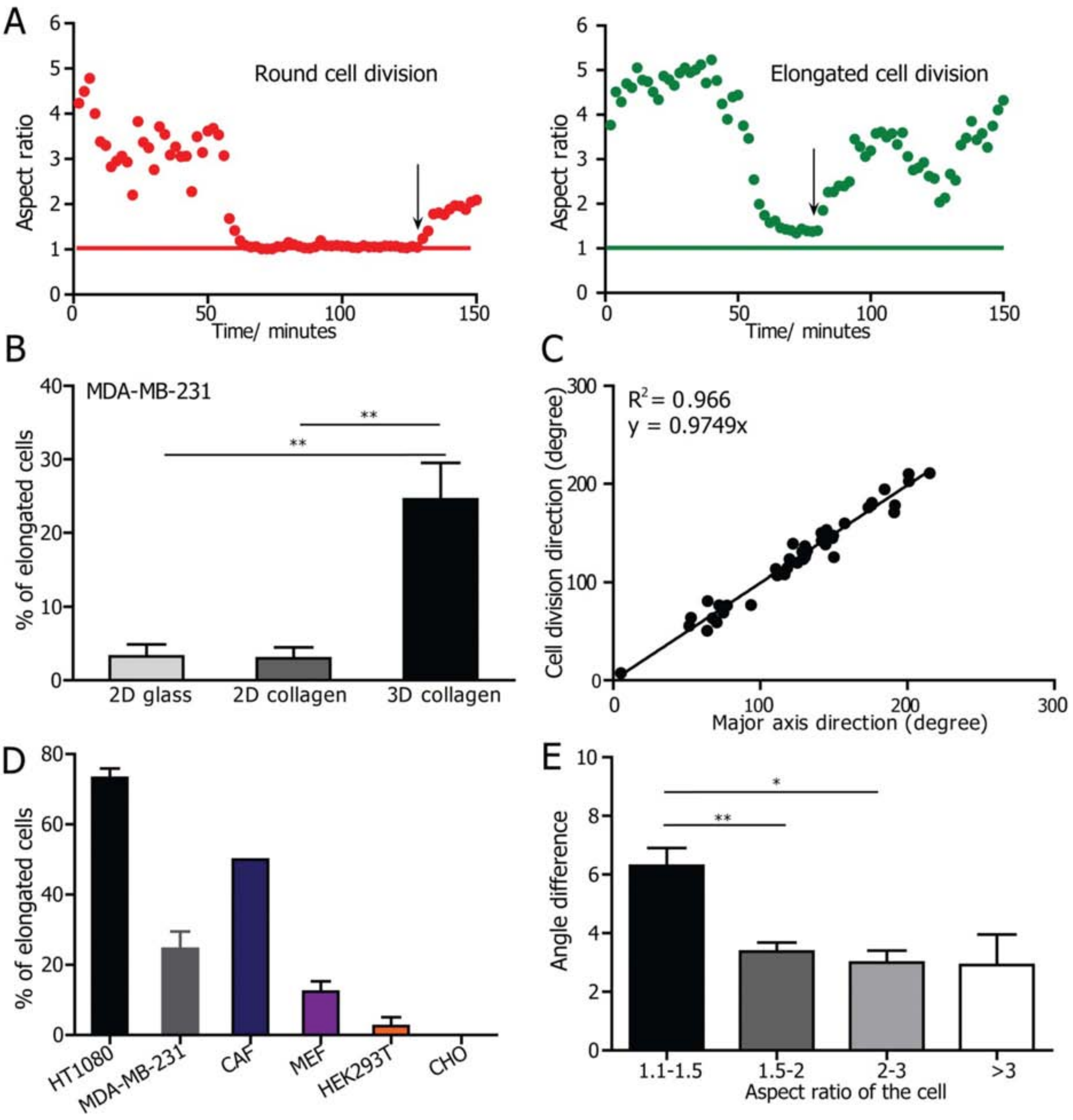

Supplementary Figure S1: Cell shape determines division orientation of elongated mammalian cells in 3D collagen. **A.** Changes in aspect ratios of the cells before and during round cell division (left) and elongated cell division (right). Black arrows indicate the time of cell division. The aspect ratio of the cell was calculated as (length of long axis) / (length of short axis). **B.** Percentages of MDA-MB-231 cells displaying an elongated morphology at mitotic phase on 2D glass with or without collagen I coating, and embedded inside a 2 mg/ml 3D collagen I matrix. $n = 3$. Data are represented as mean ± SEM. **C.** Correlation between the direction of the major axis and the direction of division for elongated MDA-MB-231 cells embedded in 3D collagen matrices. **D.** The elongated division mode of a few other types of cells, including mouse embryonic fibroblasts cells, cancer associated fibroblast cells, human embryonic kidney (HEK) 293T cells, and Chinese hamster ovary (CHO) cells, **E.** The deviation of the division direction from the long axis rule as a function of the aspect ratio of the cell before division.

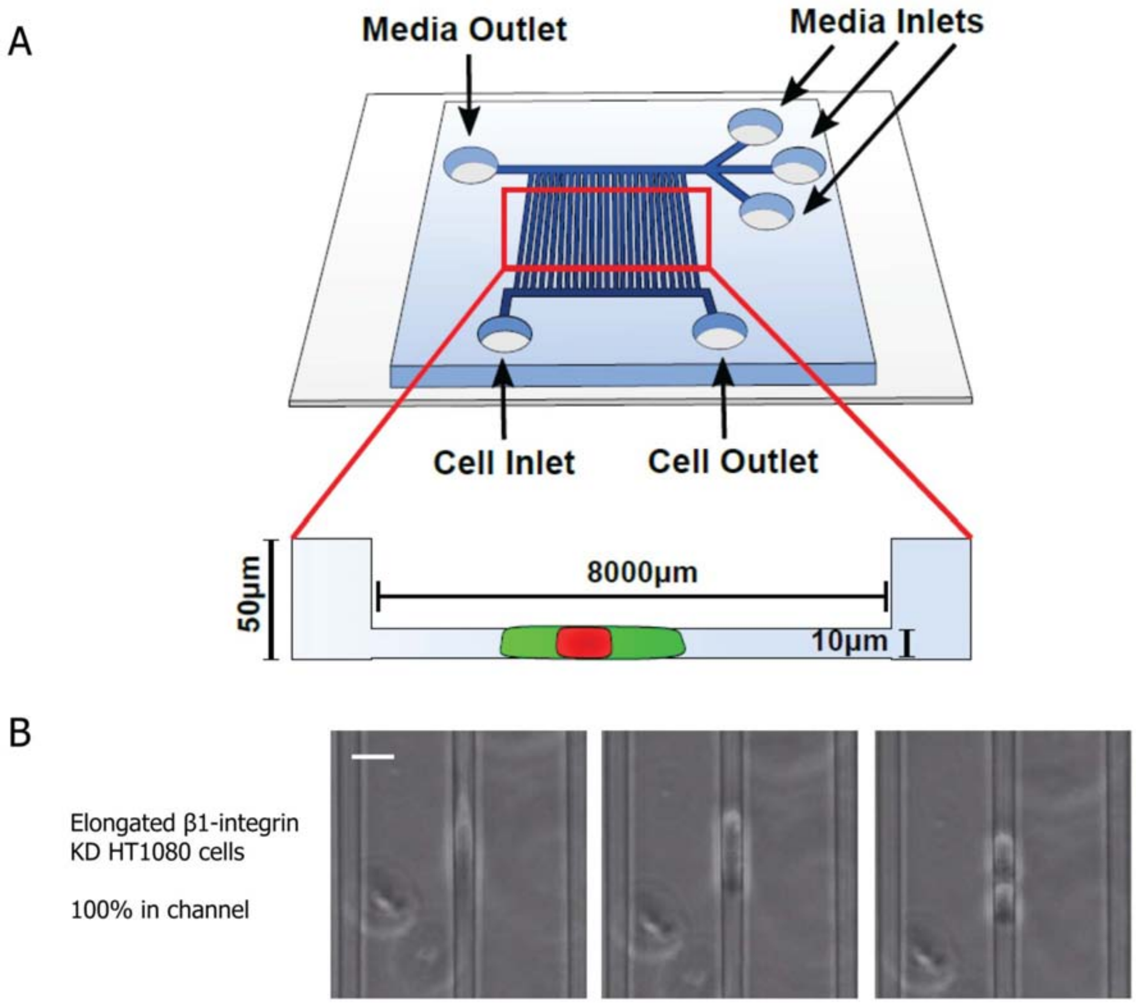

Supplementary Figure S2: The elongated mode of cell division can be recapitulated using narrow microfabricated microchannels. A. Schematic of the microfluidic-based microchannel assay to study confined cell division. PDMS microchannel device with designated cell and media inlet and outlet wells (light blue), made from standard replica molding from silicon wafers with defined channel features previously fabricated with multilayer photolithography, was bonded irreversibly to a piece of glass slide (light grey) after plasma treatment and coated with 20 µg/ml type 1 collagen. HT1080 cells were seeded in the devices and allowed to migrate into the channels with a chemotactic gradient (10% FBS to top right media inlet and serum-free media to bottom two media inlets and the cell outlet wells) for 5 hours before the media in all 6 wells were changed to 10% FBS containing media and live time-lapse imaging. The microchannels, as shown in the close up, were 12 µm or 100–200 µm in width, 10 µm in height and 8000 µm in length. **B.** All the β1-integrin-KD HT1080 cells (n = 34) divided following the elongated division phenotype when placed in the 12 µm microchannel.

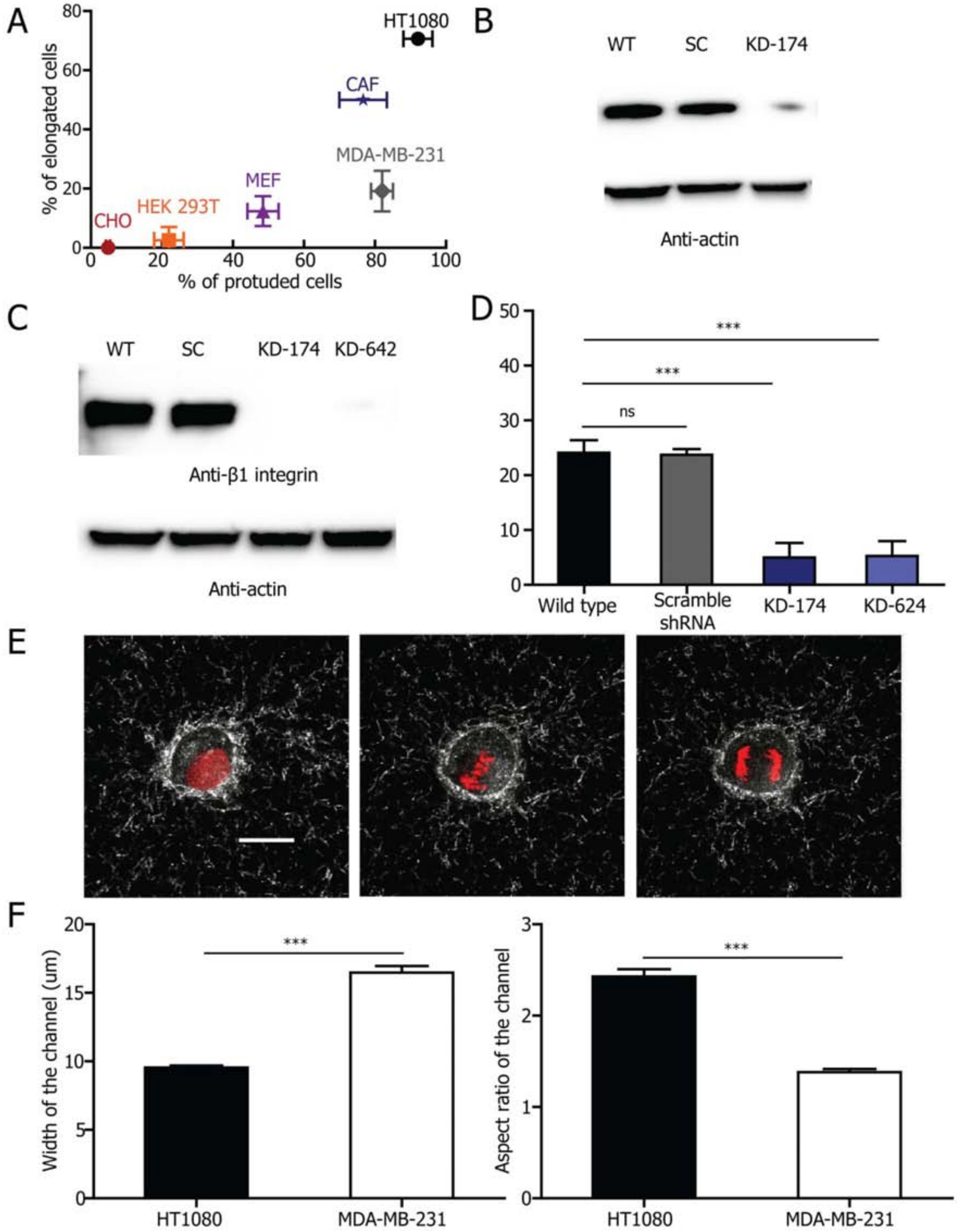

Supplementary Figure S3: Elongated cell-division phenotype depends on β1 intergrin. A. Positive correlation between the fraction of cells undergoing elongated cell division and the fraction of cells that are protruded at interphase in 3D collagen matrix. **B.** Western blotting showing the successful depletion of β1 integrin in HT1080 by shRNA segment. Cells treated by scramble shRNA served as control. **C.** Western blotting showing the successful depletion of β1 integrin in MDA-MB-231 cells by two different shRNA segments. **D.** Fraction of MDA-MB-231 cells undergoing elongated cell division in 1.5 mg/ml collagen matrix after depletion of β1 integrin. $n = 3$. Data are represented as mean ± SEM. **E.** Isotropic confinement of collagen fibers on a round-dividing β1-integrin knock-down HT1080 cell in collagen matrix. **F.** Quantification of the width and the aspect ratio of the channel generated by MDA-MB-231 cells. The data was analyzed from 387 HT1080 and 84 MDA-MB-231 from three independent experiments. Data are represented as mean ± SEM.

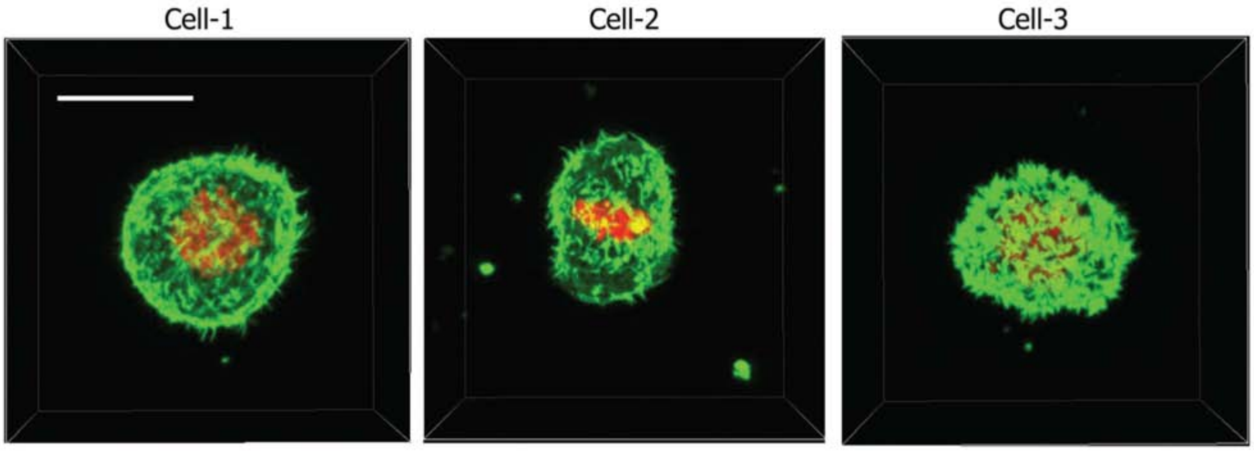

Supplementary Figure S4: Representative images of elongated cells stably expressing Lifeact-EGFP and H2B-mCherry at metaphase, which showed the absence of retraction fibers in the mitotic cell embedded in 3D collagen matrices. The fluorescence images were scanned using a Nikon A1 confocal microscope with a 60x water-immersion lens, NA = 1.2, WD = 200 μm (Nikon) and controlled by Nikon Elements imaging software (NIS-3.1). A *z*-step of 0.3 μm was used to optically section the samples and reconstructed using the "volume view" function in the Nikon Elements imaging software to obtain the 3D information of the mitotic cell.

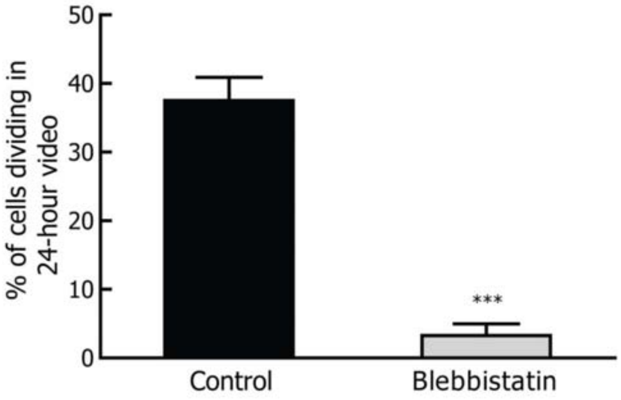

Supplementary Figure S5: Effect of blebbistatin treatment on cell division rate of HT1080 cells in collagen matrix. 50 μM blebbistatin was added to cells embedded in collagen matrix. The control cells were embedded in collagen matrices supplemented with 0.1% DMSO. *n* = 3. Data are represented as mean ± SEM.

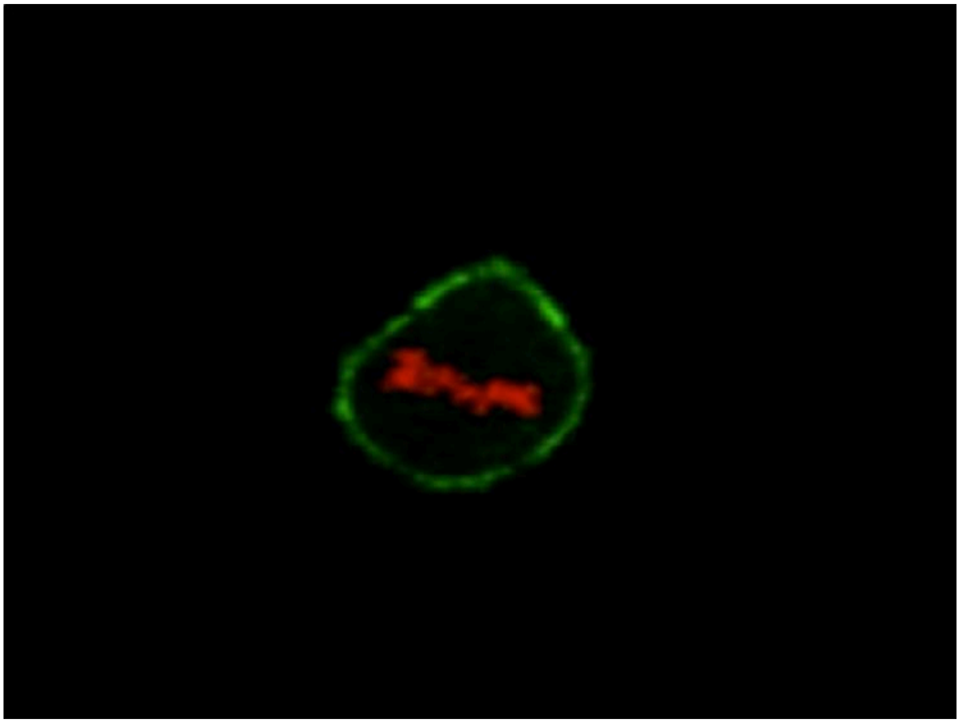

Supplementary Video-1: High magnification live-cell imaging of a matrix-embedded HT1080 cell stably expressing Lifeact-EGFP and H2B-mCherry. The whole video spans 10 hours.

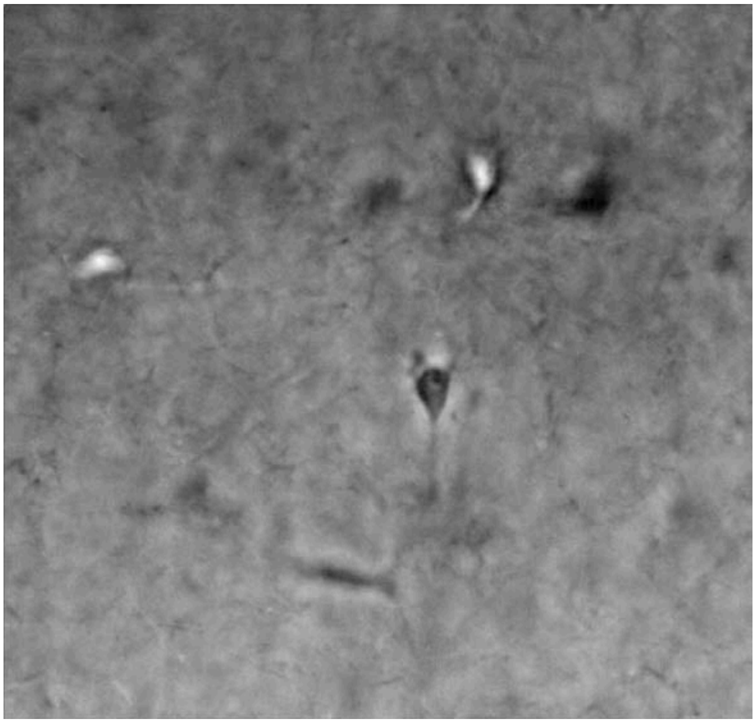

Supplementary Video-2: High magnification reflection confocal microscopy & live-cell imaging of a matrix-embedded HT1080 cell stably expressing Lifeact-EGFP and H2B-mCherry. The collagen I fibers are visualized by time-dependent reflection confocal microscopy. The whole video spans 10 hours.

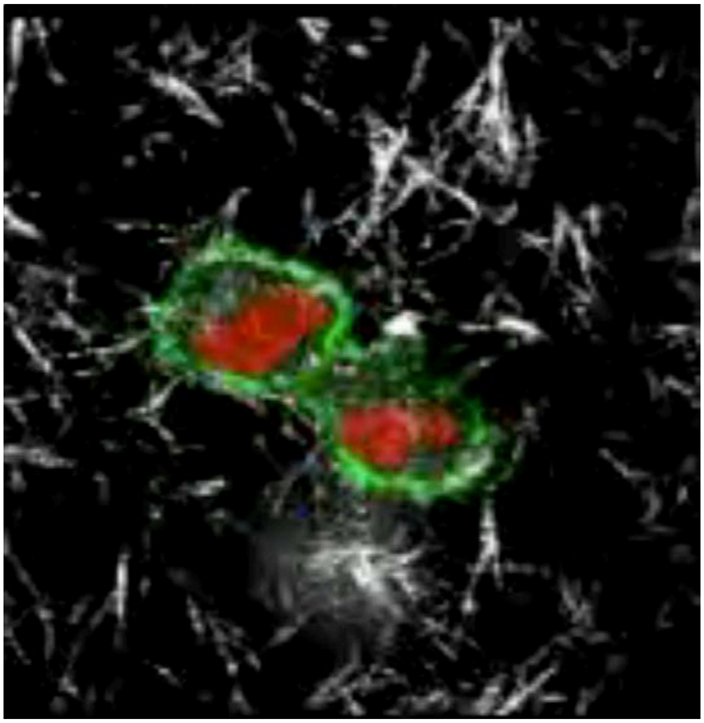

Supplementary Video-3: Elongated division of a β1 integrin KD HT1080 cell in microchannel of 12 µm in width. The video spans two and half hours.

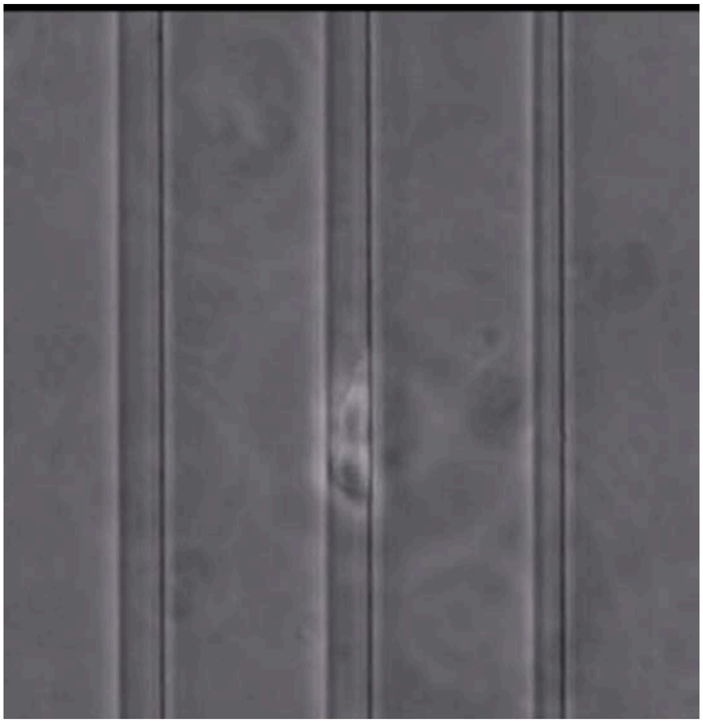

Supplementary Video-4: Elongated division of a wild type HT1080 cell in microchannel of 12 µm in width. The video spans two and half hours.

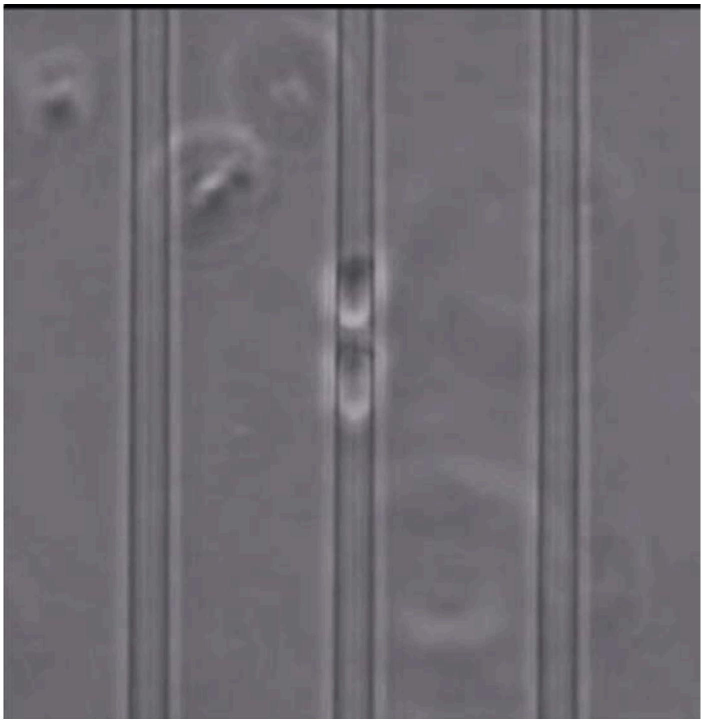

Supplementary Video-5: Division of a mother cell that underwent elongated cell division and of its two daughter cells. The video spans 40 hours.
